# Reflection on Statistical Innovations in The Past 25 Years of Alzheimer’s Disease Research

**DOI:** 10.1002/alz70859_100008

**Published:** 2025-12-25

**Authors:** Craig Mallinckrodt, Suzanne B. Hendrix, Kent Hendrix, Samuel P. Dickson

**Affiliations:** ^1^ Pentara Corporation, Salt Lake City, UT USA

## Abstract

**Background:**

The foundational statistical challenge faced in the past 25 years of Alzheimer’s Disease (AD) research stems from the shift to longer duration trials in early disease stage patients from shorter duration trials in later disease stage patients. Although beginning a disease modifying treatment in early disease is best for patients, clinical trials in early‐stage patients are problematic because disease progression is slow, and it is difficult for a treatment to slow progression much when there is not much progression to slow.

**Method:**

We compare statistical methods from 25 years ago to current methods, and then conclude with an evaluation of future directions

**Results:**

Twenty‐five years ago, Statistical Analysis Plans for AD Clinical Trial used: 1) a primary analysis based on ANCOVA and simple, ad‐hoc imputation of missing data (e.g., last observation carried forward); 2) objectives and endpoints were specified, but clear statements on exactly what was to be estimated were lacking; 3) sensitivity analyses to assess the robustness of assumptions were not clearly outlined.

Today, we have 1) multiple, principled, analytic options that can be tailored to the situation; e.g., MMRM and disease progression models; 2) clarity in what is to be estimated (estimands) and handling of intercurrent events; 3) clarity in assumptions and in the sensitivity analyses to test robustness of inferences to invalid assumptions; 4) increased precision / power via innovative designs (Bayesian adaptive dose finding, escalating cohort, sample size re‐estimation), optimized endpoints (composite or global statistical test), and principled analyses that together make informative Phase 2 trials in AD feasible; 5) reduced variability due to implementation of CDISC standard dataset structures; 6) results expressed as time saved and % slowing to understand the clinical relevance.

**Conclusions and future directions:**

Although the focus of this presentation is on clinical trials, statisticians have and will need to continue contributing to all facets of drug development, including methods to help identify better drug candidates, AI and machine learning applications, risk prediction models, new diagnostics, and fluid and digital biomarker development and validation for surrogacy. With recently approved drugs, optimized designs for clinical trials of combination treatments will be needed.

